# Virtual Interviews and the Pediatric Emergency Medicine Match Geography: A National Survey

**DOI:** 10.5811/westjem.18581

**Published:** 2024-03-14

**Authors:** Aline Baghdassarian, Jessica A. Bailey, Derya Caglar, Michelle Eckerle, Andrea Fang, Katherine McVety, Thuy Ngo, Jerri A. Rose, Cindy Ganis Roskind, Melissa M. Tavarez, Frances Turcotte Benedict, Joshua Nagler, Melissa L. Melissa L. Langhan

**Affiliations:** * Inova L.J. Murphy Children's Hospital, Department of Pediatrics, Falls Church, Virginia; † University of Virginia, School of Education, Charlottesville, Virginia; ‡ Oregon Health & Science University, Department of Pediatrics and Emergency Medicine, Portland, Oregon; § University of Washington, Department of Pediatrics, Seattle, Washington; ∥ Seattle Children’s Hospital, Department of Pediatrics, Seattle, Washington; ¶ University of Cincinnati College of Medicine, Department of Pediatrics, Cincinnati, Ohio; # Cincinnati Children’s Hospital, Department of Pediatrics, Cincinnati, Ohio; ** Stanford University School of Medicine, Department of Pediatric Emergency Medicine, Palo Alto, California; †† Children’s Hospital of Michigan, Department of Pediatrics, Detroit, Michigan; ‡‡ Central Michigan University, School of Medicine, Department of Pediatrics, Detroit, Michigan; §§ Johns Hopkins University, School of Medicine, Department of Pediatrics, Baltimore, Maryland; ∥∥ Rainbow Babies & Children’s Hospital, Department of Pediatrics, Cleveland, Ohio; ¶¶ Case Western Reserve University, School of Medicine, Department of Pediatrics, Cleveland, Ohio; ## Columbia University Irving Medical Center, Pediatrics in Emergency Medicine, New York, New York; *** University of Pittsburgh, School of Medicine, Department of Pediatrics, Pittsburgh, Pennsylvania; ††† University of Missouri of Kansas City School of Medicine, Department of Pediatrics, Kansas City, Missouri; ‡‡‡ University of Kansas Medical Center, Kansas City, Missouri; §§§ Boston Children’s Hospital, Department of Pediatrics and Emergency Medicine, Boston, Massachusetts; ∥∥∥ Harvard Medical School, Department of Pediatrics and Emergency Medicine, Boston, Massachusetts; ¶¶¶ Yale University School of Medicine, Department of Pediatrics and Emergency Medicine, New Haven, Connecticut

## Abstract

**Introduction:**

Virtual interviews (VI) are now a permanent part of pediatric emergency medicine (PEM) recruitment, especially given the cost and equity advantages. Yet inability to visit programs in person can impact decision-making, leading applicants to apply to more programs. Moreover, the cost advantages of VI may encourage applicants to apply to programs farther away than they might otherwise have been willing or able to travel. This could create unnecessary strain on programs. We conducted this study to determine whether PEM fellowship applicants would apply to a larger number of programs and in different geographic patterns with VI (2020 and 2021) as compared to in-person interviews (2018 and 2019).

**Methods:**

We conducted an anonymous national survey of all PEM fellows comparing two cohorts: current fellows who interviewed inperson (applied in 2018/2019) and fellows who underwent VIs in 2020/2021 (current fellows and those recently matched in 2021). The study took place in March–April 2022. Questions focused on geographic considerations during interviews and the match. We used descriptive statistics, chi-square and *t*-tests for analysis.

**Results:**

Overall response rate was 42% (231/550); 32% (n = 74) interviewed in person and 68% (n = 157) virtually. Fellows applied to a median of 4/6 geographic regions (interquartile range 2, 5). Most applied for fellowship both in the same region as residency (216, 93%) and outside (192, 83%). Only the Pacific region saw a statistically significant increase in applicants during VI (59.9% vs 43.2%, *P* = 0.02). There was no statistical difference in the number of programs applied to during in-person vs VI (mean difference (95% confidence interval 0.72, −2.8 – 4.2). A majority matched in their preferred state both during VI (60.4%) and in-person interviews (65.7%). The difference was not statistically significant (*P* = 0.45).

**Conclusion:**

While more PEM fellowship applicants applied outside the geographic area where their residency was and to the Pacific region, there was no overall increase in the number of programs or geographic areas PEM applicants applied to during VI as compared to in-person interview seasons. As this was the first two years of VI, ongoing data collection will further identify trends and the impactof VI.

What do we already know about this issue?

*Virtual interviews are a permanent part of recruitment. They offer cost and equity advantages while posing challenges to both applicants and programs.*

What was the research question?

*Did PEM fellowship applicants apply to a larger number of programs and in different geographic patterns with VI as compared to in-person interviews?*

What was the major finding of the study?

*VI did not have a significant impact on the number of programs or geographic areas applicants applied to.*

## INTRODUCTION

Since 2020, virtual interviews (VI) have been preferred for trainee recruitment.^1^ With the benefits of lower cost and greater equity, it is likely to remain a permanent part of recruitment, despite a general preference for face-to-face interviews.^2^ ^–^ ^5^ The VI process and associated perceptions have been described in the literature.^2^ ^,^ ^3^ ^,^ ^6^ ^–^ ^9^ The inability to visit a program in person can impact decision-making during ranking,^4^ ^,^ ^10^ ^–^ ^14^ and an increased number of applications could create undue strain on programs.^15^ ^–^ ^17^

Geographic location, sense of “fit,” and program leadership were described as major contributors to applicants’ rank preference.^18^ A national cohort of pediatric emergency medicine program directors (PEM PD), in a joint statement, raised concern that VI could lead applicants to apply to more programs and to programs farther away than they may be willing or able to travel.^10^ We conducted this study to determine whether PEM fellowship applicants would apply to a larger number of programs and in different geographic patterns with VI (2020 and 2021) as compared to in-person interviews (2018 and 2019).

## METHODS

### Design and Participants

This was an anonymous, self-administered, cross-sectional, web-based survey of PEM fellows in the United States. Participation was voluntary, and no incentive was provided for completion. The study was exempted by the institutional review board at Yale University, with informed consent implied by completion of the survey by participants.

### Survey Development

The survey questionnaire was developed through iterative feedback and a modified Delphi process to determine item importance. Thirteen PEM PDs with expertise in performance and evaluation participated in multiple rounds of revisions and editing. Pilot testing was conducted with two pediatric hospital medicine fellows who had applied to the match during VIs and two pediatric chief residents who were also interviewing for fellowships using VI, at the lead institution. Revisions were made based on pilot feedback (survey provided in Supplementary Appendix 1). The survey included multiple-choice questions about location of residency, states applied to and interviewed for fellowship, preferred location for fellowship, states visited in person for the purpose of the match, and state matched in. It also asked fellows to indicate states of residence of immediate family (parents, siblings, or partners) and about compelling reasons (other than family) that may have led fellows to favor a state or region (free text). Geographic regions were defined as Northeast, Southeast, Midwest, Southwest, Rocky Mountain, and Pacific regions.^19^

### Survey Distribution

The survey was reviewed and approved by the American Academy of Pediatrics (AAP) Section on Emergency Medicine (SOEM) PD survey subcommittee prior to distribution on Qualtrics (Qualtrics, Provo, UT) to all PEM PDs, via the AAP SoEM PD Committee listserv. The PDs forwarded the survey link to their current and incoming fellows (those recently matched to start in July 2022). Each PD completed a separate questionnaire indicating the total number of current and recently matched fellows to whom they forwarded the survey.

### Analysis

Participants were divided into two groups: VI (2020 or 2021) and in person (2018 or 2019). We performed descriptive statistics including frequencies, percentages, means with standard deviations, and medians with interquartile range (IQR). Chi-square tests compared categorical variables and t-tests, continuous variables with 95% confidence intervals (CI). We considered a two-tailed alpha of <0.05 to be statistically significant. We conducted analyses in IBM SPSS Statistics version 28 (IBM Corporation, Armonk, NY).

## RESULTS

The PDs reported that they forwarded the survey to 406 current fellows and 144 incoming fellows. The response rate for current fellows was 35% (143/406) and for incoming fellows, 61% (88/144). Overall, the response rate was 42% (231/550). Of the total respondents, 62% (143/231) were current fellows and 38% (88/231) incoming. Two fellows (1%) did not complete residency in the US, and 12 (5%) applied to PEM fellowship more than once.

All incoming fellows had undergone VI, whereas 48% of the current fellows had undergone VI (69/143). Overall, 32% of respondents (74/231) interviewed in person and 68% (157/213) virtually. There was no statistical difference in the number of programs applied to during in-person vs VI (mean difference (95% CI): .72 [−2.8, 4.2]) (Appendix 2 Table).

Data describing the geographic training and location preference of participants are presented in the table in appendix 2. Fellows applied to a median of four of the six geographic regions (IQR 2, 5). Most participants applied for fellowship in the same geographic region as their residency (216, 93%) and outside their residency region as well (192, 83%). Only the Pacific region saw a statistically significant increase in applicants during VI (59.9% vs 43.2%, *P* = 0.02) (Table 1).

**Table 1.** Influence of virtual interviews on applicant behavior and outcomes.

	In-person interviews (N = 74)	Virtual interviews(N = 157)	Statistical significance (*P* value or 95% CI)
Applied to region for fellowship, N (%)			
Northeast	59 (79.7)	123 (78.3)	0.81
Southeast	41 (55.4)	102 (65)	0.16
Midwest	50 (67.6)	111 (70.7)	0.63
Southwest	38 (51.4)	86 (54.8)	0.63
Rocky Mountains	31 (41.9)	73 (46.5)	0.51
Pacific	32 (43.2)	94 (59.9)	0.02
Applied to same geographic region as residency, N (%)	71 (98.6)	145 (94.8)	.278
Applied outside geographic region as residency, N (%)	56 (77.8)	136 (88.9)	0.03
Number of regions applied to, mean (SD)	3.4 (1.8)	3.8 (1.8)	Mean difference (95% CI): .36 (−.15, .89)
Number of states applied to, mean (SD)	9 (7.3)	9.7 (6.8)	Mean difference (95% CI): .73 (−1.2, 2.7)
Number of programs applied to, mean (SD)	13.3 (12.8)	14 (12.5)	Mean difference (95% CI): .72 (−2.8, 4.2)
Number of programs interviewed at, mean (SD)	7.2 (4.7)	6.9 (5.2)	Mean difference (95% CI): −3.1 (−1.7, 1.1)
Matched in preferred state, N (%)	46 (65.7)	84 (60.4)	0.46
Matched in same state as residency, N (%)	31 (42%)	59 (38%)	0.58
Preferred to match in state with immediate family present, N (%)	36 (52.9)	59 (46.8)	0.42
Went to visit state/program, N(%)	9 (14)	23 (17)	0.61

*CI*, confidence interval.

Less than half of respondents had immediate family members living in the same state as residency (N = 111, 48%), fellowship (N = 90, 39%), or their preferred match state (N = 95, 41%). Compelling reasons to apply to an area included familiarity with location (N = 128, 55%); similar location to residency (N = 65, 28%); and a desire to train in a new area (N = 53, 23%). Partner’s employment was an important factor for 89 (38%), salary and cost of living for 76 (33%), and school for children for 20 (9%).

## DISCUSSION

Our results show that VI may allow some candidates to explore and consider regions they may not have otherwise due to logistical or financial constraints, without increasing the number of programs, regions or states they apply to. These results are consistent with the 2021 NRMP survey where 52% reported no impact of the VI on the number of programs applied to.^5^ Residency programs have reported an increase in matched internal candidates during VI.^11^ ^,^ ^12^ ^,^ ^20^ ^,^ ^21^ In PEM, a pre-pandemic study of PDs showed that 29% of fellows completed residency at the same institution.^22^ While we did not have data at the institutional level, there was no significant increase in fellows matching within the state of their residency program with VI. This suggests that VI were not a significant detriment to applicants ranking programs and geographic areas, despite the absence of opportunities to meet in person and visit programs. This also allows programs to have access to a larger and potentially more diverse pool of candidates.^9^

Proximity to family was not a significant consideration for most applicants and was not impacted by VI. Residency applicants reported geography, quality of life, case variety, curriculum, institutional reputation, expertise in areas of interest, and program size as key factors.^23^ Applicants to PEM highlighted familiarity with the region or wanting to explore a new area as factors for exploring programs in different regions.

## LIMITATIONS

Limitations of this study include the smaller response rate of the current fellows as compared to the incoming fellows. This low response rate limited the sample size of the in-person cohort, impacting the statistical significance of our results. This differential response from the incoming fellows may have been due to desirability bias where this cohort of applicants may have tended to state that they matched in their preferred state. To minimize this, we designed our study to be fully anonymous and self-administered, and the questions were worded to retain objectivity of the answers. Respondents may also have experienced recall bias regarding the states and programs to which they applied. This bias could potentially have contributed to the lower response rate among the current fellows who had interviewed in 2018/2019, 3–4 years prior to the survey date, compared to the more recent applicants who had a more recent recollection of the questions asked in the survey.

Another limitation is that we didn’t explicitly ask the total number of fellows in each class cohort; however, since the PEM fellowship class size in the US doesn’t vary significantly from year to year (by virtue of the approved fellowship positions available), the denominator is expected to be relatively constant.

This study was not designed to look at the rates of applications to individual programs nor assess the post-match opinions of programs and fellows regarding the results of the match. This information would provide a deeper insight into the impact of the recruitment process; however, it is also prone to bias as fellows only experience training at a single institution. We also did not take into consideration the concentration of PEM programs by region or the available fellowship slots per program or region. However, the objective of this study was to look at the differences before and during VIs, and there was not a significant change in available fellowship slots or programs during these years. As the number of pediatric fellowship applicants rises, further investigation into the impact of VIs is necessary to gain a deeper understanding of its implications and to optimize this process both for applicants and programs.^24^

## CONCLUSION

While more PEM fellowship applicants applied outside the geographic area where their residency was and to the Pacific region, there was no overall increase in the number of programs or geographic areas that PEM applicants applied to during VI during the first two years of its institution, as compared to in-person interview seasons. Ongoing monitoring of the interview and match seasons will help identify future trends and impact of VIs.

## Supplementary Information



