# Supplementary material for: Virtual Interviews and the Pediatric Emergency Medicine Match Geography: A National Survey
Source: West J Emerg Med. 2024 Mar 14;25(2):186–90. doi: 10.5811/westjem.18581 (PMC11000550; doi:10.5811/westjem.18581)
Supplement: Supplementary file 1 [file wjem-25-186-s001.docx]

Geography and the Match

Start of Block: Demographics

Q4 Did you complete your RESIDENCY in the United States?

- Yes (1)
- No (2)

Display This Question:

If Did you complete your RESIDENCY in the United States? = Yes

Q5 In what STATE did you complete your medical training?

▼ Alabama (1) ... Wyoming (51)

Q7 Does your immediate family live within the United States?

- Yes (4)
- No (5)

Display This Question:

If Does your immediate family live within the United States? = Yes

Q6 In which STATE(S) does your immediate family live (defined as your parents, siblings or those of your partner)?

- Alabama (1)
- Alaska (2)
- Arizona (3)
- Arkansas (4)
- California (5)
- Colorado (6)
- Connecticut (7)
- Delaware (8)
- District of Columbia (48)
- Florida (9)
- Georgia (10)
- Hawaii (11)
- Idaho (12)
- Illinois (13)
- Indiana (14)
- Iowa (15)
- Kansas (16)
- Kentucky (17)
- Louisiana (18)
- Maine (19)
- Maryland (20)
- Massachusetts (21)
- Michigan (22)
- Minnesota (23)
- Mississippi (24)
- Missouri (25)
- Montana (26)
- Nebraska (27)
- Nevada (28)
- New Hampshire (29)
- New Jersey (30)
- New Mexico (31)
- New York (32)
- North Carolina (33)
- North Dakota (34)
- Ohio (35)
- Oklahoma (36)
- Oregon (37)
- Pennsylvania (38)
- Rhode Island (39)
- South Carolina (40)
- South Dakota (41)
- Tennessee (42)
- Texas (43)
- Utah (44)
- Vermont (45)
- Virginia (46)
- Washington (47)
- West Virginia (49)
- Wisconsin (50)
- Wyoming (51)

Q47 In which year did/will you start PEM fellowship?

- 2022 (1)
- 2021 (2)
- 2020 (3)
- 2019 (4)

Q8 Which state did you MATCH in?

▼ Alabama (1) ... Wisconsin (36)

Q10 Which state did you PREFER to match in (#1 on rank list)?

▼ Alabama (1) ... Wisconsin (36)

End of Block: Demographics

Start of Block: Default Question Block

Q2 In which areas of the country did you apply to PEM fellowship?

- Image:Northeast2 (1)
- Image:Southeast2 (2)
- Image:Midwest (3)
- Image:Southwest (4)
- Image:Rockymountains (5)
- Image:Pacific (6)

Display This Question:

If In which areas of the country did you apply to PEM fellowship? = Image:Southeast2

Q1 In which of these Southeastern States did you apply to PEM Fellowship programs?

|  | Dislike (1) | Neutral (2) | Like (3) |
| --- | --- | --- | --- |
| Alabama (3) |  |  |  |
| Georgia (4) |  |  |  |
| Mississippi (5) |  |  |  |
| Florida (7) |  |  |  |
| North Carolina (8) |  |  |  |
| So Carolina (9) |  |  |  |
| Tennessee (10) |  |  |  |
| Kentucky (11) |  |  |  |
| Virginia (12) |  |  |  |
| Arkansas (15) |  |  |  |


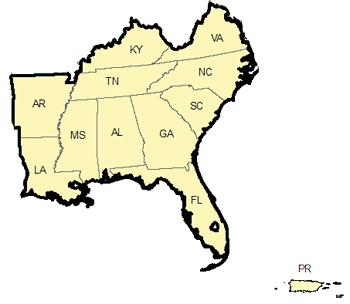


Display This Question:

If In which of these Southeastern States did you apply to PEM Fellowship programs? = Florida [ Like ]

Q3 In Florida, how many programs did you:

- Apply to (1) ________________________________________________
- Receive an Invitation to Interview (2) ________________________________________________
- Actually Interview at (3) ________________________________________________

Display This Question:

If In which of these Southeastern States did you apply to PEM Fellowship programs? = Georgia [ Like ]

Q11 In Georgia, how many programs did you:

- Apply to (1) ________________________________________________
- Receive an Invitation to Interview (2) ________________________________________________
- Actually Interview at (3) ________________________________________________

Display This Question:

If In which of these Southeastern States did you apply to PEM Fellowship programs? = Alabama [ Like ]

Q12 In Alabama, how many programs did you:

- Apply to (1) ________________________________________________
- Receive an Invitation to Interview (2) ________________________________________________
- Actually Interview at (3) ________________________________________________

Display This Question:

If In which of these Southeastern States did you apply to PEM Fellowship programs? = Arkansas [ Like ]

Q25 In Arkansas, how many programs did you:

- Apply to (1) ________________________________________________
- Receive an Invitation to Interview (2) ________________________________________________
- Actually Interview at (3) ________________________________________________

Display This Question:

If In which of these Southeastern States did you apply to PEM Fellowship programs? = Mississippi [ Like ]

Q13 In Mississippi, how many programs did you:

- Apply to (1) ________________________________________________
- Receive an Invitation to Interview (2) ________________________________________________
- Actually Interview at (3) ________________________________________________

Display This Question:

If In which of these Southeastern States did you apply to PEM Fellowship programs? = Virginia [ Like ]

Q24 In Virginia, how many programs did you:

- Apply to (1) ________________________________________________
- Receive an Invitation to Interview (2) ________________________________________________
- Actually Interview at (3) ________________________________________________

Display This Question:

If In which of these Southeastern States did you apply to PEM Fellowship programs? = North Carolina [ Like ]

Q47 In North Carolina, how many programs did you:

- Apply to (1) ________________________________________________
- Receive an Invitation to Interview (2) ________________________________________________
- Actually Interview at (3) ________________________________________________

Display This Question:

If In which of these Southeastern States did you apply to PEM Fellowship programs? = So Carolina [ Like ]

Q48 In South Carolina, how many programs did you:

- Apply to (1) ________________________________________________
- Receive an Invitation to Interview (2) ________________________________________________
- Actually Interview at (3) ________________________________________________

| Page Break |  |
| --- | --- |

Display This Question:

If In which areas of the country did you apply to PEM fellowship? = Image:Northeast2

Q15 In which of these Northeastern States did you apply to PEM Fellowship programs?

|  | Dislike (1) | Neutral (2) | Like (3) |
| --- | --- | --- | --- |
| New York (15) |  |  |  |
| Pennsylvania (16) |  |  |  |
| New Jersey (17) |  |  |  |
| Connecticut (18) |  |  |  |
| Rhode Island (19) |  |  |  |
| Massachussetts (20) |  |  |  |
| Delaware (21) |  |  |  |
| Maryland (22) |  |  |  |
| District of Columbia (23) |  |  |  |


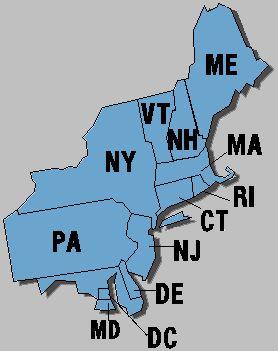


Display This Question:

If In which of these Northeastern States did you apply to PEM Fellowship programs? = New York [ Like ]

Q16 In New York, how many programs did you:

- Apply to (1) ________________________________________________
- Receive an Invitation to Interview (2) ________________________________________________
- Actually Interview at (3) ________________________________________________

Display This Question:

If In which of these Northeastern States did you apply to PEM Fellowship programs? = Pennsylvania [ Like ]

Q17 In Pennsylvania, how many programs did you:

- Apply to (1) ________________________________________________
- Receive an Invitation to Interview (2) ________________________________________________
- Actually Interview at (3) ________________________________________________

Display This Question:

If In which of these Northeastern States did you apply to PEM Fellowship programs? = New Jersey [ Like ]

Q18 In New Jersey, how many programs did you:

- Apply to (1) ________________________________________________
- Receive an Invitation to Interview (2) ________________________________________________
- Actually Interview at (3) ________________________________________________

Display This Question:

If In which of these Northeastern States did you apply to PEM Fellowship programs? = Connecticut [ Like ]

Q19 In Connecticut, how many programs did you:

- Apply to (1) ________________________________________________
- Receive an Invitation to Interview (2) ________________________________________________
- Actually Interview at (3) ________________________________________________

Display This Question:

If In which of these Northeastern States did you apply to PEM Fellowship programs? = Rhode Island [ Like ]

Q20 In Rhode Island, how many programs did you:

- Apply to (1) ________________________________________________
- Receive an Invitation to Interview (2) ________________________________________________
- Actually Interview at (3) ________________________________________________

Display This Question:

If In which of these Northeastern States did you apply to PEM Fellowship programs? = Massachussetts [ Like ]

Q21 In Massachussetts, how many programs did you:

- Apply to (1) ________________________________________________
- Receive an Invitation to Interview (2) ________________________________________________
- Actually Interview at (3) ________________________________________________

Display This Question:

If In which of these Northeastern States did you apply to PEM Fellowship programs? = Maryland [ Like ]

Q49 In Maryland, how many programs did you:

- Apply to (1) ________________________________________________
- Receive an Invitation to Interview (2) ________________________________________________
- Actually Interview at (3) ________________________________________________

Display This Question:

If In which of these Northeastern States did you apply to PEM Fellowship programs? = District of Columbia [ Like ]

Q50 In the District of Columbia, how many programs did you:

- Apply to (1) ________________________________________________
- Receive an Invitation to Interview (2) ________________________________________________
- Actually Interview at (3) ________________________________________________

Display This Question:

If In which of these Northeastern States did you apply to PEM Fellowship programs? = Delaware [ Like ]

Q51 In Delaware, how many programs did you:

- Apply to (1) ________________________________________________
- Receive an Invitation to Interview (2) ________________________________________________
- Actually Interview at (3) ________________________________________________

| Page Break |  |
| --- | --- |

Display This Question:

If In which areas of the country did you apply to PEM fellowship? = Image:Midwest

Q26 In which of these Midwestern States did you apply to PEM Fellowship programs?

|  | Dislike (1) | Neutral (2) | Like (3) |
| --- | --- | --- | --- |
| Ohio (21) |  |  |  |
| Michigan (22) |  |  |  |
| Illinois (23) |  |  |  |
| Minnesota (24) |  |  |  |
| Missouri (25) |  |  |  |
| Wisconsin (26) |  |  |  |
| Indiana (27) |  |  |  |


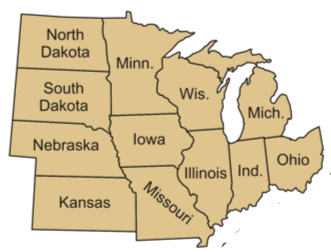


Display This Question:

If In which of these Midwestern States did you apply to PEM Fellowship programs? = Ohio [ Like ]

Q27 In Ohio, how many programs did you:

- Apply to (1) ________________________________________________
- Receive an Invitation to Interview (2) ________________________________________________
- Actually Interview at (3) ________________________________________________

Display This Question:

If In which of these Midwestern States did you apply to PEM Fellowship programs? = Michigan [ Like ]

Q28 In Michigan, how many programs did you:

- Apply to (1) ________________________________________________
- Receive an Invitation to Interview (2) ________________________________________________
- Actually Interview at (3) ________________________________________________

Display This Question:

If In which of these Midwestern States did you apply to PEM Fellowship programs? = Illinois [ Like ]

Q29 In Illinois, how many programs did you:

- Apply to (1) ________________________________________________
- Receive an Invitation to Interview (2) ________________________________________________
- Actually Interview at (3) ________________________________________________

Display This Question:

If In which of these Midwestern States did you apply to PEM Fellowship programs? = Minnesota [ Like ]

Q30 In Minnesota, how many programs did you:

- Apply to (1) ________________________________________________
- Receive an Invitation to Interview (2) ________________________________________________
- Actually Interview at (3) ________________________________________________

Display This Question:

If In which of these Midwestern States did you apply to PEM Fellowship programs? = Missouri [ Like ]

Q31 In Missouri, how many programs did you:

- Apply to (1) ________________________________________________
- Receive an Invitation to Interview (2) ________________________________________________
- Actually Interview at (3) ________________________________________________

Display This Question:

If In which of these Midwestern States did you apply to PEM Fellowship programs? = Wisconsin [ Like ]

Q32 In Wisconsin, how many programs did you:

- Apply to (1) ________________________________________________
- Receive an Invitation to Interview (2) ________________________________________________
- Actually Interview at (3) ________________________________________________

Display This Question:

If In which of these Midwestern States did you apply to PEM Fellowship programs? = Indiana [ Like ]

Q33 In Indiana, how many programs did you:

- Apply to (1) ________________________________________________
- Receive an Invitation to Interview (2) ________________________________________________
- Actually Interview at (3) ________________________________________________

| Page Break |  |
| --- | --- |

Display This Question:

If In which areas of the country did you apply to PEM fellowship? = Image:Southwest

Q34 In which of these Southern States did you apply to PEM Fellowship programs?

|  | Dislike (1) | Neutral (2) | Like (3) |
| --- | --- | --- | --- |
| Texas (28) |  |  |  |
| Arizona (29) |  |  |  |
| New Mexico (30) |  |  |  |
| Oklahoma (31) |  |  |  |


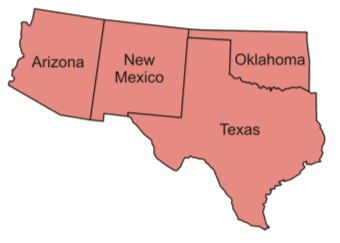


Display This Question:

If In which of these Southern States did you apply to PEM Fellowship programs? = Texas [ Like ]

Q35 In Texas, how many programs did you:

- Apply to (1) ________________________________________________
- Receive an Invitation to Interview (2) ________________________________________________
- Actually Interview at (3) ________________________________________________

Display This Question:

If In which of these Southern States did you apply to PEM Fellowship programs? = Arizona [ Like ]

Q36 In Arizona, how many programs did you:

- Apply to (1) ________________________________________________
- Receive an Invitation to Interview (2) ________________________________________________
- Actually Interview at (3) ________________________________________________

Display This Question:

If In which of these Southern States did you apply to PEM Fellowship programs? = New Mexico [ Like ]

Q37 In New Mexico, how many programs did you:

- Apply to (1) ________________________________________________
- Receive an Invitation to Interview (2) ________________________________________________
- Actually Interview at (3) ________________________________________________

Display This Question:

If In which of these Southern States did you apply to PEM Fellowship programs? = Oklahoma [ Like ]

Q38 In Oklahoma, how many programs did you:

- Apply to (1) ________________________________________________
- Receive an Invitation to Interview (2) ________________________________________________
- Actually Interview at (3) ________________________________________________

| Page Break |  |
| --- | --- |

Display This Question:

If In which areas of the country did you apply to PEM fellowship? = Image:Rockymountains

Q39 In which of these Rocky Mountain States did you apply to PEM Fellowship programs?

|  | Dislike (1) | Neutral (2) | Like (3) |
| --- | --- | --- | --- |
| Colorado (32) |  |  |  |
| Utah (33) |  |  |  |
| Nevada (34) |  |  |  |


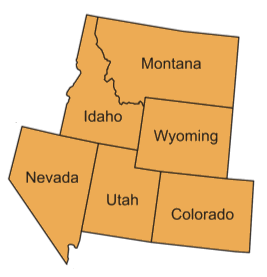


Display This Question:

If In which of these Rocky Mountain States did you apply to PEM Fellowship programs? = Colorado [ Like ]

Q40 In Colorado how many programs did you:

- Apply to (1) ________________________________________________
- Receive an Invitation to Interview (2) ________________________________________________
- Actually Interview at (3) ________________________________________________

Display This Question:

If In which of these Rocky Mountain States did you apply to PEM Fellowship programs? = Utah [ Like ]

Q41 In Utah how many programs did you:

- Apply to (1) ________________________________________________
- Receive an Invitation to Interview (2) ________________________________________________
- Actually Interview at (3) ________________________________________________

Display This Question:

If In which of these Rocky Mountain States did you apply to PEM Fellowship programs? = Nevada [ Like ]

Q42 In Nevada how many programs did you:

- Apply to (1) ________________________________________________
- Receive an Invitation to Interview (2) ________________________________________________
- Actually Interview at (3) ________________________________________________

| Page Break |  |
| --- | --- |

Display This Question:

If In which areas of the country did you apply to PEM fellowship? = Image:Pacific

Q43 In which of these Pacific States did you apply to PEM Fellowship programs?

|  | Dislike (1) | Neutral (2) | Like (3) |
| --- | --- | --- | --- |
| Washington (35) |  |  |  |
| Oregon (36) |  |  |  |
| California (37) |  |  |  |


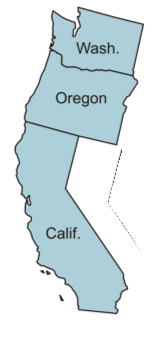


| Page Break |  |
| --- | --- |

Display This Question:

If In which of these Pacific States did you apply to PEM Fellowship programs? = Washington [ Like ]

Q44 In Washington how many programs did you:

- Apply to (1) ________________________________________________
- Receive an Invitation to Interview (2) ________________________________________________
- Actually Interview at (3) ________________________________________________

Display This Question:

If In which of these Pacific States did you apply to PEM Fellowship programs? = Oregon [ Like ]

Q45 In Oregon how many programs did you:

- Apply to (1) ________________________________________________
- Receive an Invitation to Interview (2) ________________________________________________
- Actually Interview at (3) ________________________________________________

Display This Question:

If In which of these Pacific States did you apply to PEM Fellowship programs? = California [ Like ]

Q46 In California how many programs did you:

- Apply to (1) ________________________________________________
- Receive an Invitation to Interview (2) ________________________________________________
- Actually Interview at (3) ________________________________________________

End of Block: Default Question Block

Start of Block: Block 2

Q50 Did you go on any second-looks or visit an area you were interested in?

- Yes (1)
- No (2)

Display This Question:

If Did you go on any second-looks or visit an area you were interested in? = Yes

Q49 Which state(s) did you visit?

- Alabama (1)
- Arizona (2)
- Arkansas (3)
- California (4)
- Colorado (5)
- Connecticut (6)
- Delaware (7)
- District of Columbia (35)
- Florida (8)
- Georgia (9)
- Illinois (10)
- Indiana (11)
- Kentucky (12)
- Maryland (13)
- Massachusetts (14)
- Michigan (15)
- Minnesota (16)
- Mississippi (17)
- Missouri (18)
- Nevada (19)
- New Jersey (20)
- New Mexico (21)
- New York (22)
- North Carolina (23)
- Oklahoma (24)
- Ohio (25)
- Oregon (26)
- Pennsylvania (27)
- Rhode Island (28)
- South Carolina (29)
- Tennessee (30)
- Texas (31)
- Utah (32)
- Virginia (33)
- Washington (34)
- Wisconsin (36)

Q51 Please describe any compelling reasons you had (other than family) to match in a specific area.

________________________________________________________________

________________________________________________________________

________________________________________________________________

________________________________________________________________

________________________________________________________________

End of Block: Block 2
