# Supplementary material for: Virtual Interviews and the Pediatric Emergency Medicine Match Geography: A National Survey
Source: West J Emerg Med. 2024 Mar 14;25(2):186–90. doi: 10.5811/westjem.18581 (PMC11000550; doi:10.5811/westjem.18581)
Supplement: Supplementary file 2 [file wjem-25-186-s002.docx]

Table. Geographic data of PEM fellowship applicants

| States in Region (Number of PEM Fellowship Programs in State) | State where respondent Completed Residency N (%) | State where respondent Matched for Fellowship N (%) | Matched for Fellowship in same state as Residency N (%) | Matched in Preferred State (N=209)  N (%) |
| --- | --- | --- | --- | --- |
| **Northeast Region** | 94 (40.9) | 74 (32.2) |  |  |
| Connecticut (2) | 8 (3.4) | 11 (4.8) | 4 (50) | 6 (60) |
| Delaware (1) | 1 (.4) | 4 (1.7) | 1 (100) | 0 (0) |
| District of Columbia (1) | 4 (1.7) | 0 | 0 (0) | NA |
| Maryland (1) | 3 (1.3) | 3 (1.3) | 0 (0) | 1 (50) |
| Maine (0) | 0 | NA | NA | NA |
| Massachusetts (3) | 9 (3.9) | 6 (2.6) | 3 (37.5) | 4 (80) |
| New Hampshire (0) | 0 | NA | NA | NA |
| New Jersey (2) | 2 (.9) | 0 | 0 | NA |
| New York (13) | 49 (21.1) | 30 (12.9) | 22 (44.9) | 22 (75.9) |
| Pennsylvania (3) | 16 (6.9) | 15 (6.5) | 5 (31.3) | 11 (73.3) |
| Rhode Island (1) | 2 (.2) | 5 (2.2) | 0 (0) | 1 (25) |
| Vermont (0) | 0 | NA | NA | NA |
| **Southeast Region** | 36 (15.7) | 34 (14.8) |  |  |
| Alabama (1) | 4 (1.7) | 5 (2.2) | 3 (75) | 3 (60) |
| Arkansas (1) | 0 | 0 | NA | NA |
| Florida (3) | 10 (4.3) | 5 (2.2) | 1 (10) | 1 (25) |
| Georgia (2) | 2 (.9) | 0 | 0 | NA |
| Kentucky (1) | 4 (1.7) | 5 (2.2) | 2 (50) | 3 (60) |
| Louisiana (0) | 0 | NA | NA | NA |
| Mississippi (1) | 0 | NA | NA | NA |
| No Carolina (3) | 5 (2.2) | 11 (4.7) | 3 (60) | 8 (80) |
| South Carolina (1) | 5 (2.2) | 6 (2.6) | 3 (60) | 2 (40) |
| Tennessee (2) | 2 (.9) | NA | 0 | NA |
| Virginia (3) | 3 (1.3) | 2 (.9) | 1 (33.3) | NA |
| **Midwest Region** | 50 (21.7) | 67 (29.1) |  |  |
| Illinois (2) | 6 (2.6) | 12 (5.2) | 1 (16.7) | 6 (54.5) |
| Indiana (1) | 2 (.9) | 1 (.4) | 0 (0) | 0 (0) |
| Iowa (0) | 2 (.9) | NA | NA | NA |
| Kansas (0) | 0 | NA | NA | NA |
| Michigan (4) | 3 (1.3) | 7 (3) | 1 (33.3) | 3 (50) |
| Minnesota (1) | 6 (2.6) | 6 (2.6) | 5 (83.3) | 5 (100) |
| Missouri (3) | 8 (3.4) | 14 (6) | 6 (75) | 5 (38.5) |
| Nebraska (0) | 0 | NA | NA | NA |
| N Dakota (0) | 0 | NA | NA | NA |
| Ohio (4) | 17 (7.3) | 27 (11.6) | 11 (64.7) | 17 (63) |
| S Dakota (0) | 1 (.4) | NA | NA | NA |
| Wisconsin (1) | 5 (2.2) | 0 | 0 | NA |
| **Southwest Region** | 26 (11.3) | 19 (8.2) |  |  |
| Arizona (1) | 5 (2.2) | 0 | 0 | NA |
| New Mexico (1) | 3 (1.3) | 6 (2.6) | 1 (33.3) | 2 (33.3) |
| Oklahoma (1) | 1 (.4) | 4 (1.7) | 1 (100) | 2 (66.7) |
| Texas (4) | 17 (7.3) | 9 (3.9) | 2 (11.8) | 5 (62.5) |
| **Rocky Mountain Region** | 6 (2.6) | 16 (7) |  |  |
| Colorado (1) | 2 (.9) | 5 (2.2) | 2 (100) | 4 (100) |
| Idaho (0) | 0 | NA | NA | NA |
| Montana (0) | 0 | NA | NA | NA |
| Nevada (1) | 2 (.9) | 5 (2.2) | 1 (50) | NA |
| Utah (1) | 2 (.9) | 6 (2.6) | 2 (100) | 3 (50) |
| Wyoming (0) | 0 | NA | NA | NA |
| **Pacific Region** | 18 (7.8) | 20 (8.7) |  |  |
| California (6) | 15 (6.5) | 13 (5.7) | 7 (46.7) | 10 (88.3) |
| Oregon (1) | 1 (.4) | 3 (1.3) | 1 (100) | 3 (100) |
| Washington (1) | 2 (.9) | 4 (1.7) | 1 (50) | 2 (66.7) |
